# Supplementary material for: Prepared and highly committed despite the risk of COVID-19 infection: a cross-sectional survey of primary care physicians’ concerns and coping strategies in Singapore
Source: BMC Fam Pract. 2021 Jan 16;22:22. doi: 10.1186/s12875-021-01370-7 (PMC7811343; doi:10.1186/s12875-021-01370-7)
Supplement: Supplementary file 1 — Additional file 1. [file 12875_2021_1370_MOESM1_ESM.docx]

**DEMOGRAPHICS**

| 1. **Gender** | M / F |
| --- | --- |
| 1. **Age** | - 20 – 29 years old - 30 – 39 years old - 40 – 49 years old - ≥ 50 years old |
| 1. **Race** | Chinese / Malay / Indian / Others |
| 1. **Marital status** | Single / Married / Divorced / Widowed |
| 1. **How many children do you have?**   [*Note: Skip logic; only triggered if participant selects “Married”, “Divorced” or “Widowed” in previous item*] | 0 / 1 / 2 / 3 / > 3 |
| 1. **People in household** | Staying with family / Staying alone / Others |
| 1. **If others, please specify**   [*Note: Skip logic; only triggered if participant selects “Others” in previous item*] | [*Free response item*] |
| 1. **Type of practice** | GP / Polyclinic / Family Medicine Clinic / Restructured Hospital / Others |
| 1. **Is your clinic a PHPC?**   [*Note: Skip logic; only triggered if participant selects “GP” in previous item*] | Yes / No |
| 1. **If others, please specify**   [*Note: Skip logic; only triggered if participant selects “Others” in previous item*] | [*Free response item*] |
| 1. **Years of experience** | - 1 – 5 years - 6 – 10 years - 11 – 15 years - 16 – 20 years - > 20 years |
| 1. **Highest medical qualification** | MBBS / GDFM / MMed(FM) / MCFPS / FCFPS / Others |
| 1. **If others, please specify**   [*Note: Skip logic; only triggered if participant selects “Others” in previous item*] | [*Free response item*] |

**How much do you agree with the following statements regarding the ongoing COVID-19 outbreak in Singapore?**

Responses:

Strongly Disagree – SD

Disagree – D

Agree – A

Strongly Agree – SA

Not Applicable – NA

# Work-Related Concerns

| A1. My job would put me at great exposure risk | SD / D / A / SA |
| --- | --- |
| A2. I am afraid of falling ill with COVID-19 | SD / D / A / SA |
| A3. I should not be looking after COVID-19 patients | SD / D / A / SA |
| A4. The risk I am exposed to is not acceptable | SD / D / A / SA |
| A5. I accept that the risk of contracting COVID-19 is part of my job | SD / D / A / SA |
| A6. I might look for another job because of the risks | SD / D / A / SA |
| A7. It is acceptable if my colleagues resign because of their fears | SD / D / A / SA |
| A8. I am confident my employer would look after my needs | SD / D / A / SA |

# Non-Work Concerns

| A9. People close to me would be at high risk of getting COVID-19 because of my job | SD / D / A / SA |
| --- | --- |
| A10. In particular, I would be concerned for my spouse/ partner | SD / D / A / SA / NA |
| A11. In particular, I would be concerned for my parents | SD / D / A / SA / NA |
| A12. In particular, I would be concerned for my children | SD / D / A / SA / NA |
| A13. In particular, I would be concerned for my close friends | SD / D / A / SA |
| A14. In particular, I would be concerned for my work colleagues | SD / D / A / SA |
| A15. People close to me would be worried for my health | SD / D / A / SA |
| A16. People close to me would be worried as they may get infected by me | SD / D / A / SA |

# Perceived Impact on Personal Life and Work

| A17. I would be afraid of telling my family about the risks I am exposed to | SD / D / A / SA |
| --- | --- |
| A18. People would avoid me because of my job | SD / D / A / SA |
| A19. People would avoid my family members because of my job | SD / D / A / SA |
| A20. I would avoid telling other people about the nature of my job | SD / D / A / SA |
| A21. There would be adequate staff at my workplace to handle the increased demand | SD / D / A / SA |
| A22. There would be more conflict amongst colleagues at work | SD / D / A / SA |
| A23. I would feel more stressed at work | SD / D / A / SA |
| A24. I would have an increase in workload | SD / D / A / SA |
| A25. I would have to work overtime | SD / D / A / SA |
| A26. I would have to do work not normally done by me | SD / D / A / SA |

**Preparedness for COVID-19 Outbreak**

| A27. I have received training for infection control at my workplace | SD / D / A / SA |
| --- | --- |
| A28. I have received adequate personal protective equipment training | SD / D / A / SA |
| A29. I have someone to turn to if unsure of the use of personal protective equipment | SD / D / A / SA |
| A30. My workplace has a preparedness plan for a COVID-19 outbreak | SD / D / A / SA |
| A31. My workplace is prepared for a COVID-19 outbreak | SD / D / A / SA |
| A32. I am personally prepared for a COVID-19 outbreak | SD / D / A / SA |
| A33. The is sufficient supply of personal protective equipment for use in my workplace | SD / D / A / SA |

**How effective would the following be in helping to manage your stress during the current COVID-19 outbreak?**

Responses:

Not at all Effective – 0

Mildly Effective – 1

Moderately Effective – 2

Extremely Effective – 3

# Factors that may Help Reduce Stress

| A34. Positive attitude from colleagues in your workplace | 0 / 1 / 2 / 3 |
| --- | --- |
| A35. None of your colleagues getting COVID-19 after starting strict protective measures | 0 / 1 / 2 / 3 |
| A36. Improvement in COVID-19 patients’ condition | 0 / 1 / 2 / 3 |
| A37. Clear guidelines from workplace for infection prevention | 0 / 1 / 2 / 3 |
| A38. Personal protective equipment provided to you by workplace | 0 / 1 / 2 / 3 |
| A39. Your family members or friends not getting COVID-19 | 0 / 1 / 2 / 3 |
| A40. Decrease in COVID-19 cases reported in the new | 0 / 1 / 2 / 3 |
| A41. Likelihood that you would get extra compensation for your exposure to COVID-19 | 0 / 1 / 2 / 3 |
| A42. All healthcare professionals working together on the front line | 0 / 1 / 2 / 3 |
| A43. Confidence in fellow medical staff in case you got sick from COVID-19 | 0 / 1 / 2 / 3 |
| A44. Not having to do overtime | 0 / 1 / 2 / 3 |
| A45. Sharing jokes or humour among colleagues | 0 / 1 / 2 / 3 |
| A46. Getting free meals at your workplace | 0 / 1 / 2 / 3 |

**How often have you used the following to help cope with stress during the current COVID-19 outbreak?**

Responses:

Never Used – 0

Sometimes Used – 1

Often Used – 2

Always Used – 3

**Personal Coping Strategies to Alleviate Stress**

| A47. Followed strict personal protective measures | 0 / 1 / 2 / 3 |
| --- | --- |
| A48. Kept separate clothes for work or used scrubs to minimize risk of transmission | 0 / 1 / 2 / 3 |
| A49. Considered every patient encountered as COVID-19 case and using full protective gear even if patient was COVID-19 negative | 0 / 1 / 2 / 3 |
| A50. Read about COVID-19, its prevention and mechanisms of transmission | 0 / 1 / 2 / 3 |
| A51. Avoided going out to public places to minimize COVID-19 exposure | 0 / 1 / 2 / 3 |
| A52. Did relaxation activities (e.g. involved in prayers, sports, exercise etc.) | 0 / 1 / 2 / 3 |
| A53. Talked to family and friends to relieve stress and obtain support | 0 / 1 / 2 / 3 |
| A54. Motivational self-talk to face COVID-19 outbreak with positive attitude | 0 / 1 / 2 / 3 |
| A55. Talked to fellow colleagues or other doctors to relieve stress and obtain support | 0 / 1 / 2 / 3 |
| A56. Tried to be busy at home in activities that would keep your mind away from COVID-19 | 0 / 1 / 2 / 3 |
| A57. Avoided doing overtime to reduce exposure to COVID-19 patients | 0 / 1 / 2 / 3 |
| A58. Avoided media news about COVID-19 and related fatalities | 0 / 1 / 2 / 3 |
| A59. Vented emotions by crying, screaming etc. | 0 / 1 / 2 / 3 |
